# Retrospective analysis for thirty-nine patients with solitary fibrous tumor of pleura and review of the literature

**DOI:** 10.1186/1477-7819-9-134

**Published:** 2011-10-20

**Authors:** Wei Guo, Hua-Liang Xiao, Yao-Guang Jiang, Ru-Wen Wang, Yun-Ping Zhao, Zheng Ma, Hui-Jun Niu

**Affiliations:** 1 Department of Thoracic Surgery, Institute of Surgery Research, Daping Hospital, Third Military Medical University, Changjiang Route 10#, Chongqing, PR China; 2 Department of Pathology, Institute of Surgery Research, Daping Hospital, Third Military Medical University, Changjiang Route 10#, Chongqing, PR China

**Keywords:** Solitary fibrous tumor, Pleura, Surgical treatment, Prognosis

## Abstract

**Background:**

Solitary fibrous tumor of the pleura (SFTP) is an uncommon neoplasm arising from mesenchymal cells. The aim of this study is to summarize the experience and the outcome of the surgical treatment for 39 cases of SFTP.

**Methods:**

From January 2004 to December 2008, 39 patients underwent surgical resection of SFTP in our department. All patients had clinical follow-up by the same team of surgeons. The mean follow-up was 40.3 months.

**Results:**

A local removal of the neoplasm was accomplished by video-assisted thoracic surgery (VATS) in 9 patients (group A) and by thoracotomy in 30 patients (group B) respectively. Comparing with group B, operations in group A took significantly less operative time, blood loss and spent less time in the intensive care unit and hospital. All specimens were positive for CD34 and Bcl-2. One patient developed recurrence, and the remaining 38 patients are alive and disease free at the end of follow-up.

**Conclusions:**

Malignant SFTP still had the potential recurrence. VATS represents the more acceptable choice for the selected patients with SFTP.

## Background

As a rare primary neoplasm arising from mesenchymal cells in the areolar tissue subjacent to the mesothelial-lined pleura, the solitary fibrous tumor of the pleura (SFTP) was first mentioned by Wagner in 1870 [1], and the pathologic description did not appear until 1931 [2]. Due to the rarity of SFTP, there were only fewer than 800 cases reported before 2002 [3], and the understanding of this disease still remains unclear. Moreover, controversy about the origin of this uncommon tumor led to a variety of terms applied to the tumor in earlier years, such as localized pleural mesothelioma, pleural fibroma, localized fibrous mesothelioma, submesothelial fibroma, and localized fibrous tumor [3].

SFTP is a mesenchymal tumor that tends to involve the pleura, although it has also been described in other thoracic areas (mediastinum, pericardium and pulmonary parenchyma) and in extrathoracic sites (meninges, epiglottis, salivary glands, thyroid, kidneys and breast) [3,4]. SFTP usually presents as a peripheral mass abutting the pleural surface, to which it is attached by a broad base or, more frequently, by a pedicle that allows it to be mobile within the pleural cavity [3,5,6]. Unlike mesothelioma, SFTP is not asbestos-related and is usually a benign, rarely aggressive tumor, although a small percentage of patients may develop locoregional recurrence [3,7,8].

Due to the unclear biological characteristics, accurate prediction for the clinical course of patients with SFTP still remains difficult. Herein we analyzed a consecutive series of 39 patients with SFTP who underwent surgical resection in our department, and assessed the effectiveness and necessity of surgical therapy administered in patients with SFTP, as well as clinical and pathological features and follow-up results.

## Material and methods

### Patients

From January 2004 to December 2008, a retrospective analysis was carried out of 39 patients who underwent resection of SFTP at our department. Preoperative evaluation included bronchofibroscope and chest computed tomography (CT) scan. All these patients didn't received preoperative chemoradiotherapy. The information about the age and gender of patients, course of disease, initial manifestations or symptoms, history of tobacco consumption, location and size of the lesions, surgical complication and outcome was recorded (table 1). The histological diagnosis was obtained postoperatively from the resected specimen. Postoperative death was defined as death within 30 days after surgery or before discharge from the hospital. To reconfirm the diagnosis of SFTP, the same pathologist reviewed all available histological slides. Following histological review, all slides were re-classified as benign or malignant according to the following criteria: high cellularity, presence of nuclear atypia, mitotic count of more than four mitoses per ten high-power fields (HPF), and presence of necrosis [9]. All patients had clinical follow-up by the same team of surgeons. Stable clinical outcome was also confirmed by telephoning the patients. The mean follow-up was 40.3 months (range: 30~89 months).

**Table 1:** Clinicopathological characteristics of 39 patients with SFTP

	Number of patients	Percentage (%)
Sex		
Male	27	69.2
Female	12	30.8
Age (years)		
< 40	5	12.8
40~60	28	71.8
> 60	6	15.4
Clinical presentation		
Chest symptoms	20	51.3
Achropachy	4	10.3
Pyrexia	3	7.7
Pneumonia	1	2.6
Asymptomatic	19	48.7
Location		
Right thoracx	19	48.7
paraspinal	10	25.6
paramediastinal	5	12.8
intraparenchymal	4	10.3
Left thoracx	20	51.3
paraspinal	12	30.8
paramediastinal	3	7.7
intraparenchymal	5	12.8
Diameter (cm)		
< 5.0	9	23.1
5.0~10.0	23	59.0
> 10.0	7	17.9

Total	39	100

There was significant difference in sex ratio between male and female patients, and significance can be also observed in different age group. No significant difference in the location of lesion could be observed between the right and left thoracx.

### Surgical technique

All operations were performed under two-lumens intubation and general endotracheal anesthesia. VATS is performed in the case of small and pedunculated lesions. For bulky tumor with diameter greater than 5.0 cm or SFTP with a large broad base of attachment at the parietal pleura, thoracotomy is mandatory to achieve radicality in resection. In our series, there were 30 cases with tumor greater than 5.0 cm underwent thoracotomy and accquired integrated excision. A local removal of the neoplasm was accomplished by VATS in 9 patients (group A) and by thoracotomy in 30 patients (group B) respectively. No patient underwent aggressive pneumonectomy. For obtaining histologic negative margins, fast frozen section in operation was performed. For 9 patients with tumor of 5.0 cm diameter or smaller, surgical resection was performed under video-assisted thoracic surgery (VATS). At the end of the videothoracoscopic procedure, the tumor was put in a retrieval bag and a small incision was performed, then the tumor was pulled away.

### Immunohistochemistry

Immunohistochemistry was performed on 4-μm-thick paraffin sections using the Envision TM detection system (DakoCytomation, Carpinteria, CA) and according to the manufacturer's recommendations. The following primary antibodies were used: Mouse monoclonal antibodies for CD34 (clone QBEnd/10, working dilution, Maixin Biotech, China), Ki67 (clone MIB-1, 1:200, Dako) and Bcl-2 (clone 8C8, working dilution, Maixin Biotech, China). Negative controls were performed for each case by replacing primary antibody with mouse IgG. For Ki-67, Sections from colon adenocarcinoma were used as positive controls. For Bcl-2 and CD34, tumor infiltrating lymphocytes and vascular endothelial cells served as internal positive controls respectively. As for Ki-67, the positive cells in five maximally labeled high power fields were counted, and the labeling index (LI) was expressed as the percentage of the labeled tumor cells.

### Statistical methods

The data are expressed as the mean ± SEM. The difference between means was performed with ANOVA. All statistical analyses were performed using SPSS 11.0 software (Chicago, IL, USA) and p < 0.05 was considered as statistically significant.

## Results

**Figure 1:**
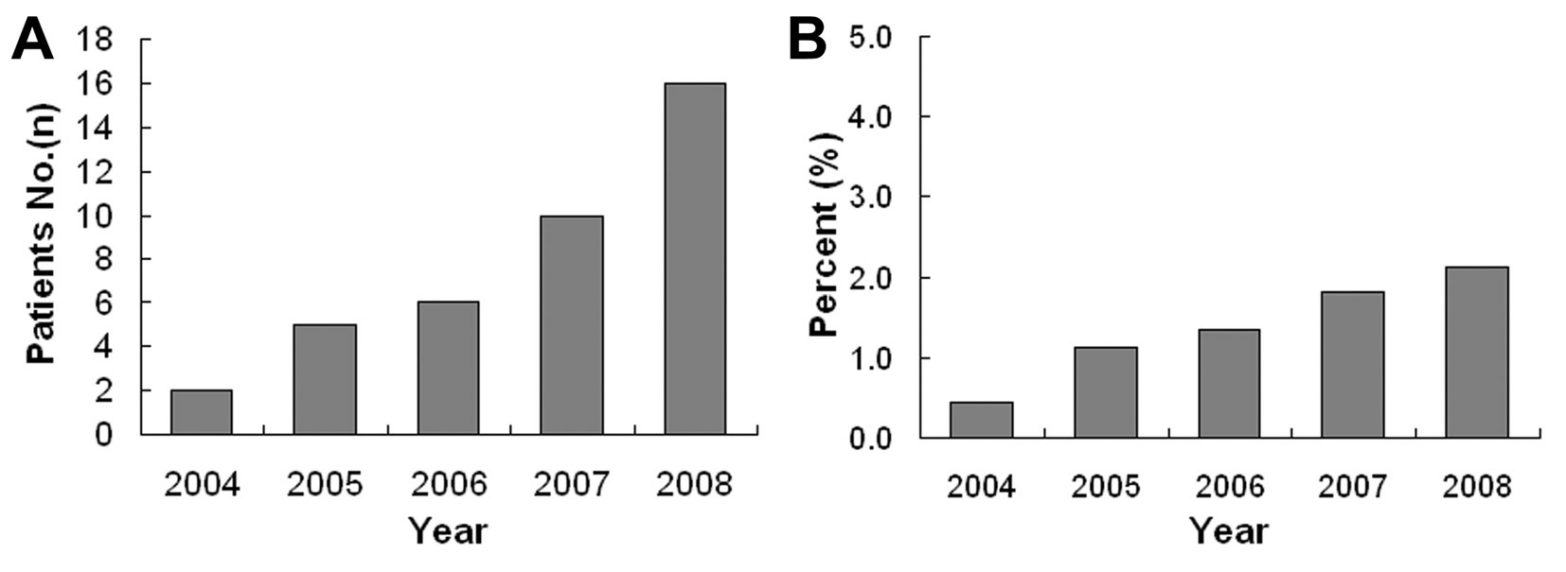
**Gradually increasing tendency of SFTPs**. (A) During the 5 years from 2004 to 2008, the cases with pathological diagnosis of SFTP in our hospital were observed gradually increased. (B) Comparing to the simultaneous total number of operations, the ratio is also in increasing.

During the 5 years from 2004 to 2008, the cases with SFTP in our hospital were observed gradually increased per year (Figure 1A). Comparing to the simultaneous total number of operations, the ratio also shows an increasing tendency (Figure 1B). In our series, 27 (69.2%) patients were female and the sex ratio (female/male) was 2.25/1. The mean age at surgery was 44.6 ± 14.5 years (range: 22~77 years). Twenty-eight of these patients (71.8%) were between 40~60 years and only 5 patients (12.8%) were under 40 (table 1). And SFTP occurred most frequently in patients aged from 40 to 60 years in our series. The course of disease ranged from 0.5 month to 6 years (mean of 1.56 years). Twenty (51.3%) of our patients had symptoms such as coughing, expectoration and chest pain. In these 20 patients, achropachy was encountered in 4 cases (10.3%), pyrexia in 3 (7.7%). One (2.6%) obstructive pneumonia due to obstruction of the bronchus was encountered. And hypoglycemia was not observed in our series. The remaining 19 patients (48.7%) were asymptomatic at the time of diagnosis (table 1). Two patients had multiple hepatic cysts and one had the history of thoracic injury. No concomitant malignant lesion was encountered.

**Figure 2:**
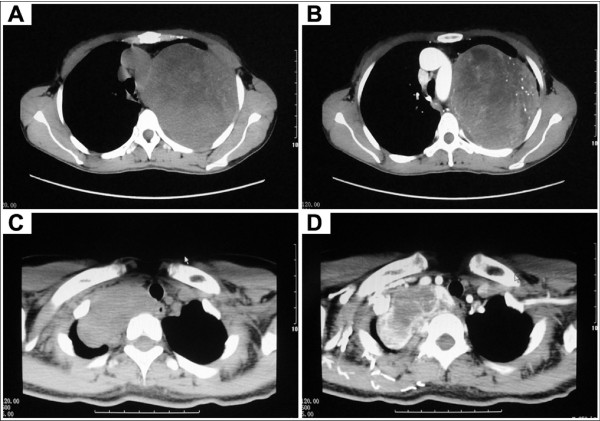
**Enhancement of SFTP showed by chest CT scans**. (A, B) Before the injection of contrast agent. (C, D) Partial enhancement of SFTP after injection.

To exclude other lessions arising from bronchus, bronchoscopic studies were performed in fifteen patients (38.5%). Twelve patients (30.8%) underwent preoperative CT-guided fine needle aspiration biopsy (FNAB). Only two specimens (16.7%) revealed benign appearing spindle cells and this suggested diagnosis of benign pleural fibrous tumor. The remaining ten cases (83.3%) specimens were not significant. Chest radiographs and CT scan examination were performed preoperatively in all cases. Chest radiographs of SFTP usually demonstrated a sharply marginated globular mass. Mostly, the manifestation of CT scan was a smoothly marginated, elliptical, abnormal mass in the thorax. The lesion has a density greater than simple fluid and with partial enhancement occasionally (Figure 2). Positron emission tomography (PET) scanning was performed in 2 cases, and the tumors had no fluorodeoxyglucose (FDG) uptake (SUV 2.1 and 1.0 respectively).

Twenty-four tumors (61.5%) arose from the visceral pleura and 15 (38.5%) from the parietal pleura, sixteen (41.0%) were pedunculated and 23 (59.0%) presented a broad base of attachment. Among tumors arising from the visceral pleura, two showed a prevalent intrapulmonary growth (the so-called inverted fibroma). All tumors arising from the parietal pleura were broad based and not pedunculated. The mean maximum diameter of tumors was 7.71 ± 4.78 cm (range: 2.0 to 22.0 cm). Most of the SFTPs in our series (59.0%) had the maximum diameter ranged from 5.0 to 10.0 cm. Nine of them (23.1%) were under 5.0 cm and 7 (17.9%) over 10.0 cm (table 1). The smallest tumor was 2.0 cm in greatest diameter and the largest was 22.0 × 19.0 × 19.0 cm. No synchronous SFTP or associated malignant disease was observed in our series.

Thirty patients underwent surgery by standard thoracotomy (group B). And VATS was performed in 9 patients (group A). Of these 9 underwent VATS, tumor resection with negative incision margin were performed and none required a conversion to open thoracotomy. Four wedge resections were performed for tumors related to the visceral pleura (group A). And two SFTPs located centrally in the lobe required lobectomy due to intraparenchimal extension of the tumor (inverted fibroma). No patient suffered from more aggressive pneumonectomy or chest-wall resection in our series. The patients' data are also given in table 2. Comparing with thoracotomy group, operations in group A took significantly less time and blood loss (114.78 ± 30.04 min and 133.33 ± 79.06 ml respectively). Althrough there was not significantly difference, the VATS group had fewer requirements for transfusion during the admission. As for the length of hospital stay, the VATS group spent less time in the intensive care unit and hospital compared with the thoracotomy approaches. The overall complication rate was not different for each group. This number includes two cases of postoperative pleural effusions (all in group B), which did not have a substantive impact on overall recovery. The incidence of respiratory infections was not different for two groups nor was the need to return to the intensive care unit with respiratory compromise. The operative mortality was not observed in both groups.

**Table 2:** Operative data of 39 patients with SFTP

	Total (n = 39)	Group A (n = 9)	Group B (n = 30)	*p*
Blood loss (ml)	308.97 ± 167.75	133.33 ± 79.06	361.67 ± 150.68	0.0001
Surgical time (min)	169.63 ± 50.28	114.78 ± 30.04	177.67 ± 50.81	0.0033
Transfusion (no. of patients)	5(5/39)	0(0/9)	5(5/30)	0.2284
LOS ICU (days)	2.77 ± 0.67	2.22 ± 0.44	3.20 ± 0.55	0.0006
LOS* (days)	14.26 ± 2.26	12.89 ± 2.20	14.67 ± 2.14	0.0262
Postoperative complications				
Pleural effusion	2(2/39)	0	2(2/30)	0.4419
Pulmonary infection	4(4/39)	1(1/9)	4(4/30)	0.8722

LOS, length of hospital stay.

**Figure 3:**
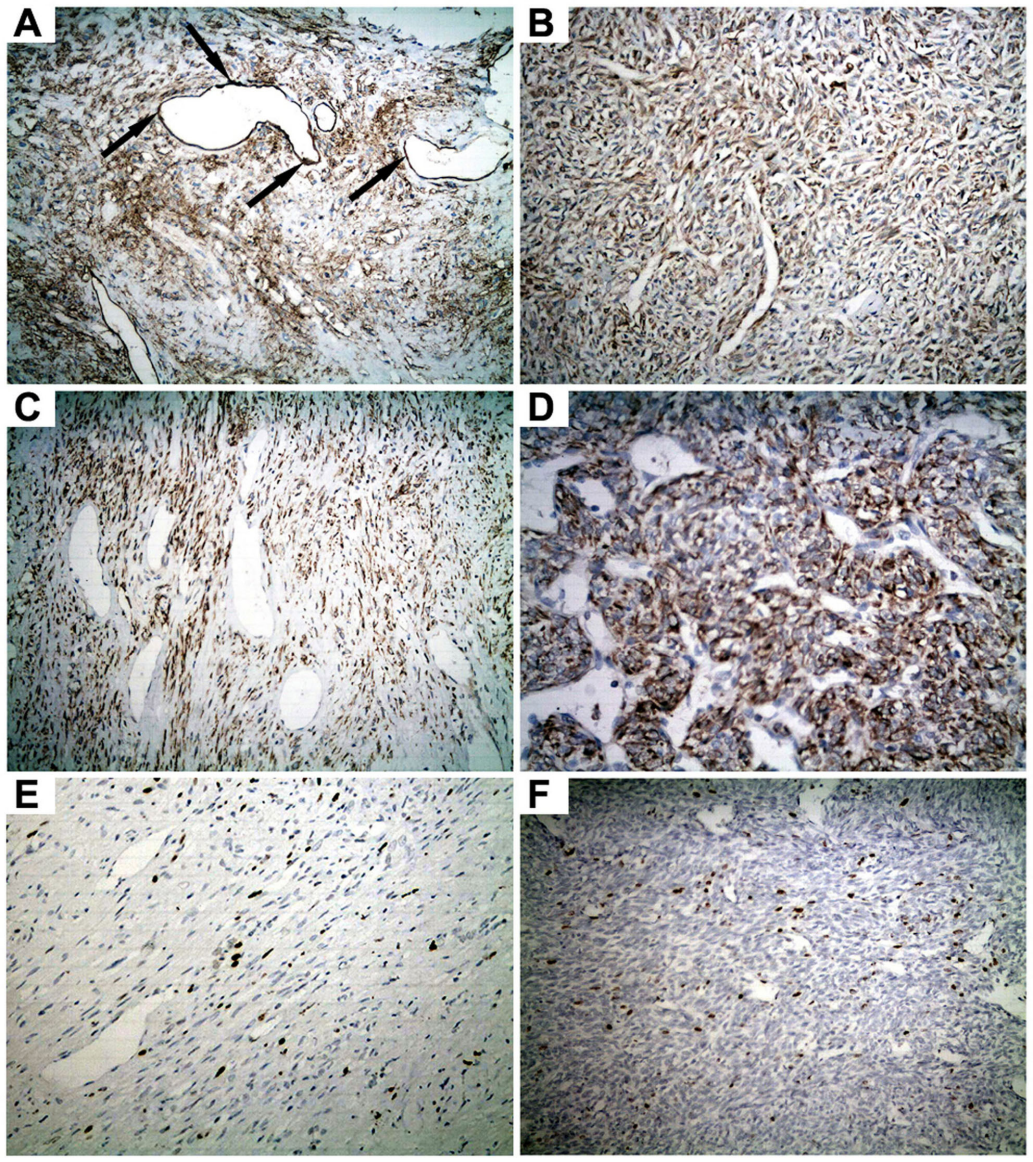
**Immunohistochemical staining of the SFTPs**. (A) Positive staining of CD34 was observed especially in the vascular endothelial cells and tumor cells (200). (B) Positive staining for CD34 in a malignant SFTP (200). (C) Strong positive expression of Bcl-2 was observed in the cytoplasm of tumor cells (200). (D) Positive staining of Bcl-2 in a malignant SFTP (200). (E) Ki-67 LI is about 5% in a benign SFTP (200). (F) A malignant case in which the Ki-67 LI is about 15% (200).

According to the criteria of England et al [9], 35 cases (89.7%) in our series were classified as benign and 4 cases (10.3%) were found to be malignant. All specimens were positive for CD34 (which helps to differentiate SFT from mesothelioma [10]). And all cases were also positive for Bcl-2. Ki-67 LI ranged from 2% to 10% and 15% to 30% in benign and malignant SFTPs respectively (Figure 3).

**Figure 4:**
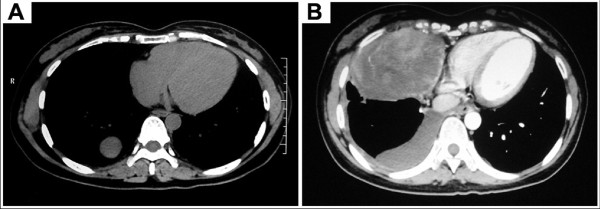
**Recurrence of SFTP in a case**. (A) CT scan showed a prevalent intrapulmonary growth (the so-called inverted fibroma). (B) Another smoothly marginated abnormal mass occurred at right thorax with pleural effusion 6 months after surgery.

Follow-up continued until June 2011, ensuring a minimal follow-up of 30 months. Thirty-eight patients submitted the regular follow-up program that included clinical examination and chest roentgenogram after 1 month and every 3 months. Chest CT scan was performed every 6 months. Local recurrence at a thoracoscopic site was not observed in our series. One patient with malignant SFTP underwent wedge resection by VATS lost during follow-up. In operation, the histologic negative margin was obtained. However, CT scan showed another smoothly marginated abnormal mass occurred at right thorax (Figure 4) when he came back 6 months after surgery. FNAB revealed spindle cells and thus suggested the recurrence of SFTP. This patient refused further treatment and lost touch again after discharging. The remaining 38 patients were submitted a mean follow-up of 40.3 months (range: 30~89 months). At the end of follow-up, none of these patients died, and the concomitant malignant lesion was not found in our series after operation.

## Discussion

According to the review by de Perrot et al [3], approximately 800 cases of SFTP have been reported in the literature before 2002 July. After carefully searching the subsequent literatures, the author observed that there were additional 760 cases of SFTP reported in the last 7 years. The increasing occurence of SFTP might owe to the advancement of methods for diagnosis, especially by the aids of immunohistochemistry and electronmicroscope. Meanwhile, this phenomenon making us hypothesesed that the incidence of SFTP seems to be increasing, and the results from our series also supported this viewpoint. An accredited hypothesis about the aetiopathogenesis of SFTP is that these tumors originate from submesothelial stromal cells with fibroblastic or myofibroblastic phenotype, whose growth is promoted by an aberrant reaction to inflammatory or hormonal stimuli [11].

By the aids of imageological examination, SFTP is relatively easy to diagnose when located in typical sites [12]. Rarely, diagnosis of SFTP may prove more challenging when the lesion is located in an unusual site. The clinical value of biopsy before excision is still controversial. Scarsbrook and his colleagues recounted an alarming case in which a solitary fibrous pleural tumor recurred after an ultrasoundguided transthoracic biopsy [13]. Although there have been no other similar reports in the literature, it seems prudent to avoid unnecessary biopsy to prevent this potential complication. As the study recommends [13], biopsy should only be done if disease management will be substantially affected by the results or if surgical intervention is contraindicated and a diagnosis would alter treatment. Among the 12 patients underwent preoperative FNAB in our series, only two cases (16.7%) specimens revealed benign appearing spindle cells and suggested diagnosis of benign pleural fibrous tumor. The remaining ten cases (83.3%) specimens were not significant. The results also demonstrated the limited assistance of preoperative biopsy for the diagnosis of SFTP.

SFTP is usually histologically benign, but the presence of cellular atypia, necrosis, overlapping of nucleus and a high mitotic index (defined as being in excess of 4/10 HPF) is strongly indicative of malignancy [9]. The neoplastic cells are typically CD34+ and BCL2+ and are negative for cytokeratin [14-16]. Occasionally, malignant SFTP exhibited a negative reaction to CD34 immunostaining [17]. But in our series, we did not observed the negative CD34 immunostaining even in malignant cases.

According to the report from Cardillo et al [18], the malignant tumors were usually greater than 10 cm in diameter. However, there were 8 cases with tumor lager than 10 cm in our series were diagnosed as benign SFTP and free of recurrence during follow-up. Therefore, the author presumed that the size of tumor might not the mandatory diagnostic criteria of malignant SFTPs. For instance, due to lack of the chance for health examination, patients from the region with poor medical condition usually have the bulky tumor.

Complete surgical resection was the preferred therapy for both benign and malignant SFTPs, and the most important indicator of the clinical outcome in SFTP is the complete and radical resection of the tumor [19]. Since this tumor is not a primary lung neoplasm, the surgeon should strive to save as much lung as possible in both the benign and malignant varieties while obtaining histologic negative margins, if the tumor arises from the visceral pleura [20]. It also stated that though pedunculated tumors are effectively removed using wedge resection, sessile lesions require that a larger mass of lung parenchyma be excised to reduce the likelihood of recurrence. In our series, local removal was the main choice for patients with SFTP. For tumors located in the pulmonary parenchyma, wedge resection or lobectomy was the preferred procedure.

Small pedunculated tumors located on the visceral pleura can be safely removed by VATS [18,21]. And some authors have also recommended the VATS to obtain a more precise view of the resection margins in some large, broad-based tumors of the parietal pleura [18]. In our opinion, the VATS is useful in the case of small and pedunculated lesions. For bulky tumor or SFTP with a large broad base of attachment at the parietal pleura, thoracotomy is mandatory to achieve radicality in resection. To avoid potential implantation metastasis, the tumor should be resected integratedly as far as possible. Moreover, because of the potential metastasis and local recurrence at the port sites, the contact between the tumor and the thoracoscopic sites also should to be avoided [18]. Therefore, we choosed the pedunculated lesion with diameter smaller than 5.0 cm as the indication of VATS. Due to the malignant variety of SFTPs, the spillage at the time of surgery should be avoided to reduce the possibility of local recurrence.

## Conclusion

In summary, although the complication did not present significance between VATS and open group, the patients underwent VATS did obtain less blood loss and operative time, as well as ICU and hospital stay. Moreover, local recurrence at a thoracoscopic site was not observed in patients underwent tumor resection under VATS. These results presented that local removal by VATS with intraoperative assessment of free surgical margins was the recommended choice for surgical treatment of selected patients with SFTP.

## Abbreviations

SFTP: Solitary fibrous tumor of the pleura; VATS: Video-assisted thoracic surgery; CT: Computed tomography; HPF: High-power fields; LI: labeling index; FNAB: Fine needle aspiration biopsy; PET: Positron emission tomography; FDG: Fluorodeoxyglucose; LOS: Length of hospital stay.

## Competing interests

The authors declare that they have no competing interests.

## Authors' contributions

WG coordinated the project, assisted in review and collection of the clinical data and drafted the manuscript. HLX carried out the immunoassays and assisted in review and collection of the clinical data. YGJ designed, coordinated the study, carried out the extraction of data, performed critical appraisal of the literature and assisted in writing the manuscript. RWW participated in the design of the study and performed the statistical analysis. YPZ, ZM, HJN conceived of the study, and participated in its design and coordination and helped to draft the manuscript. All authors read and approved the final manuscript.
